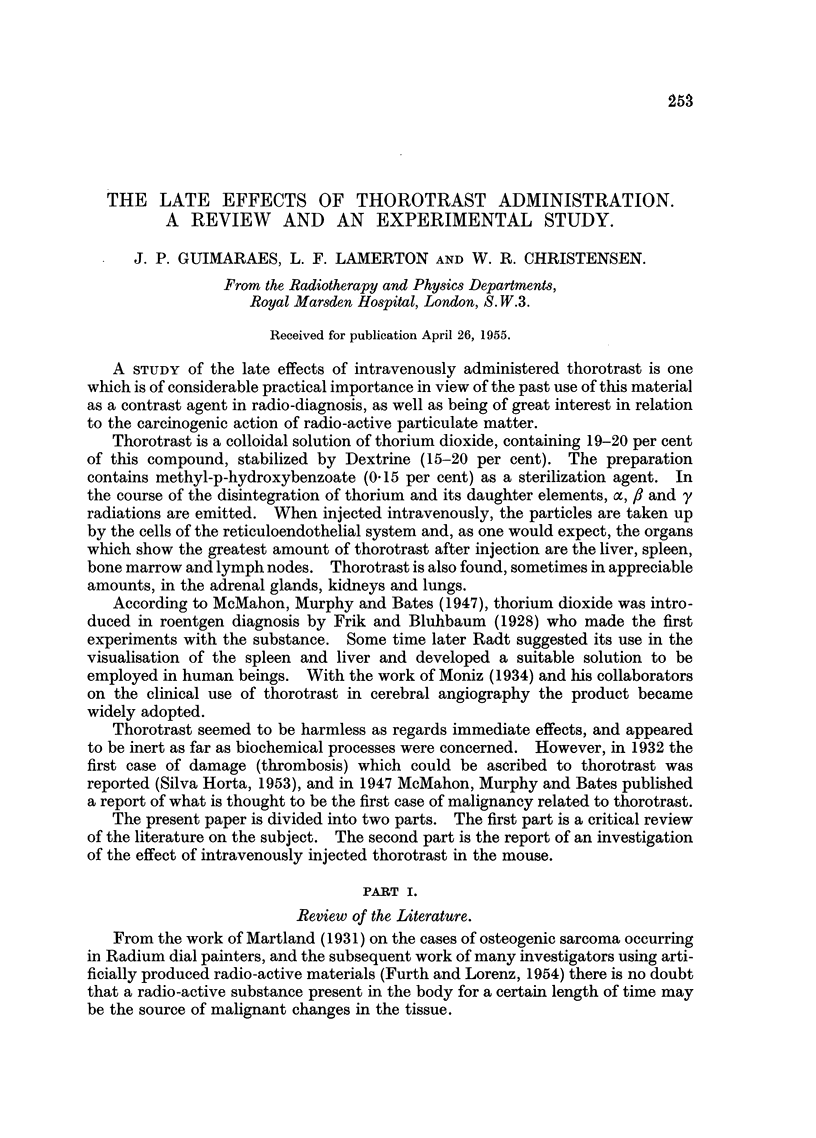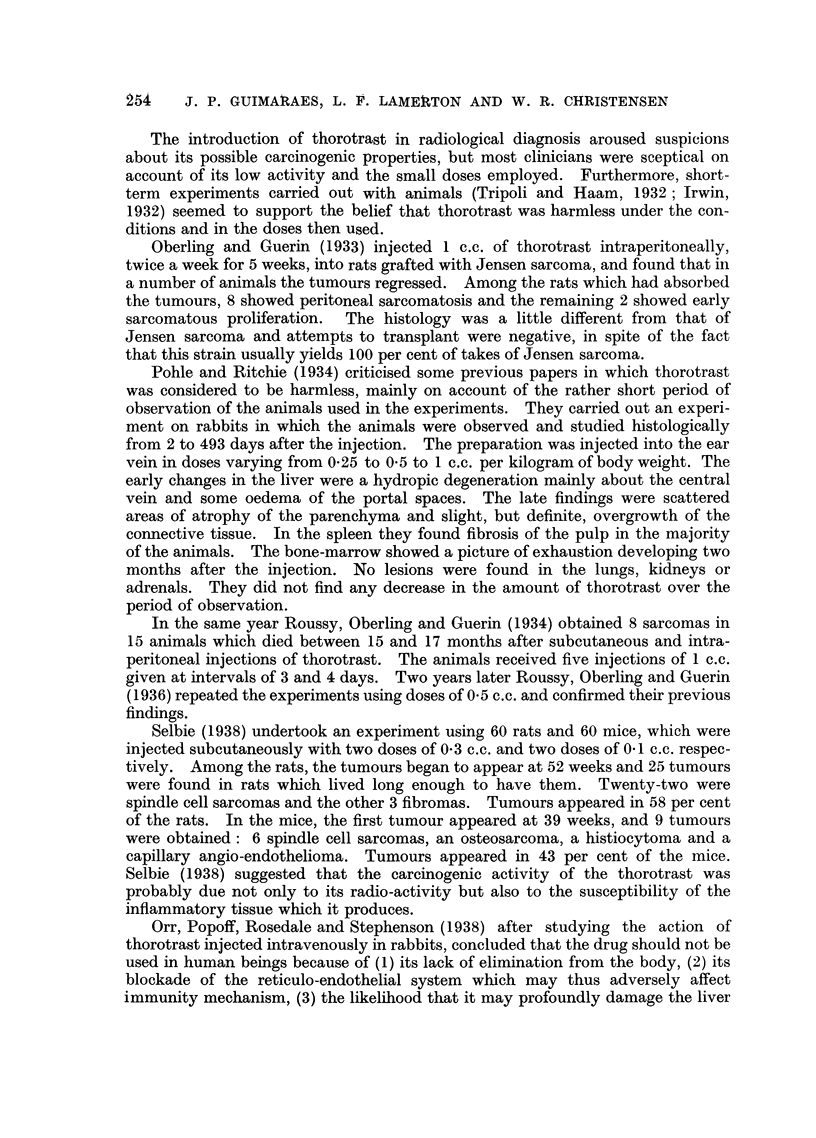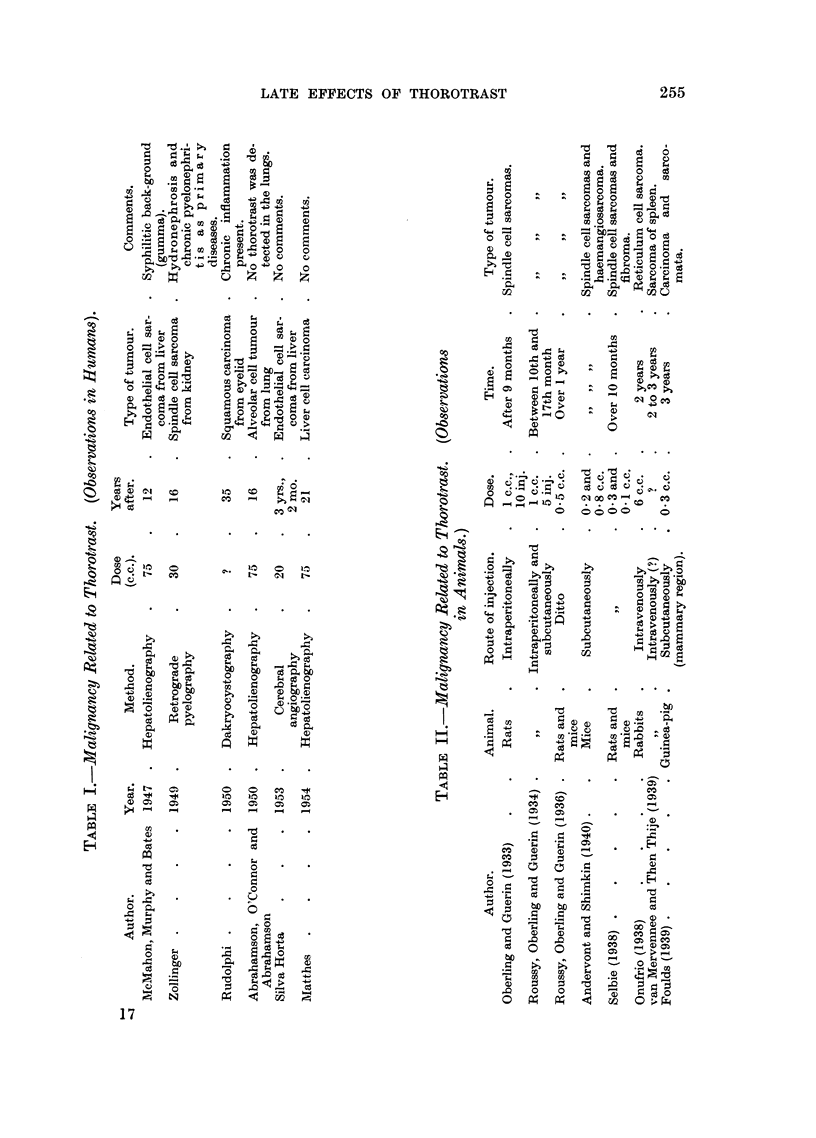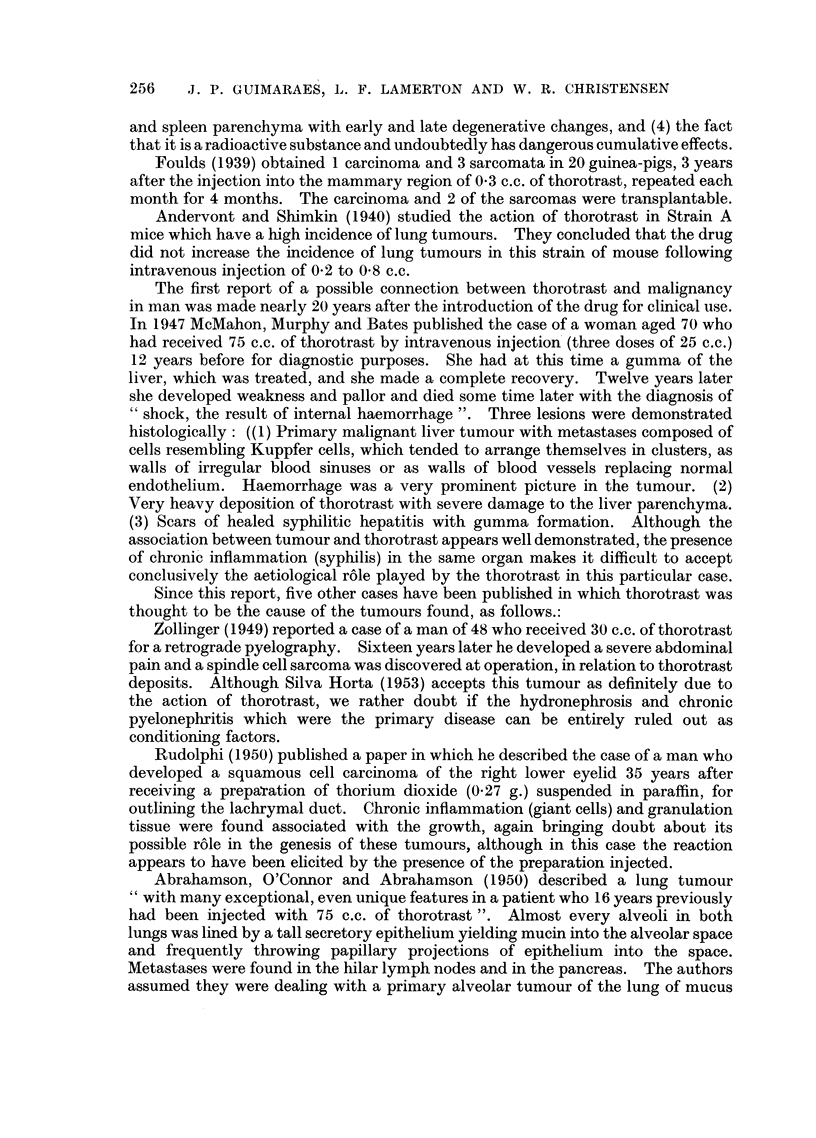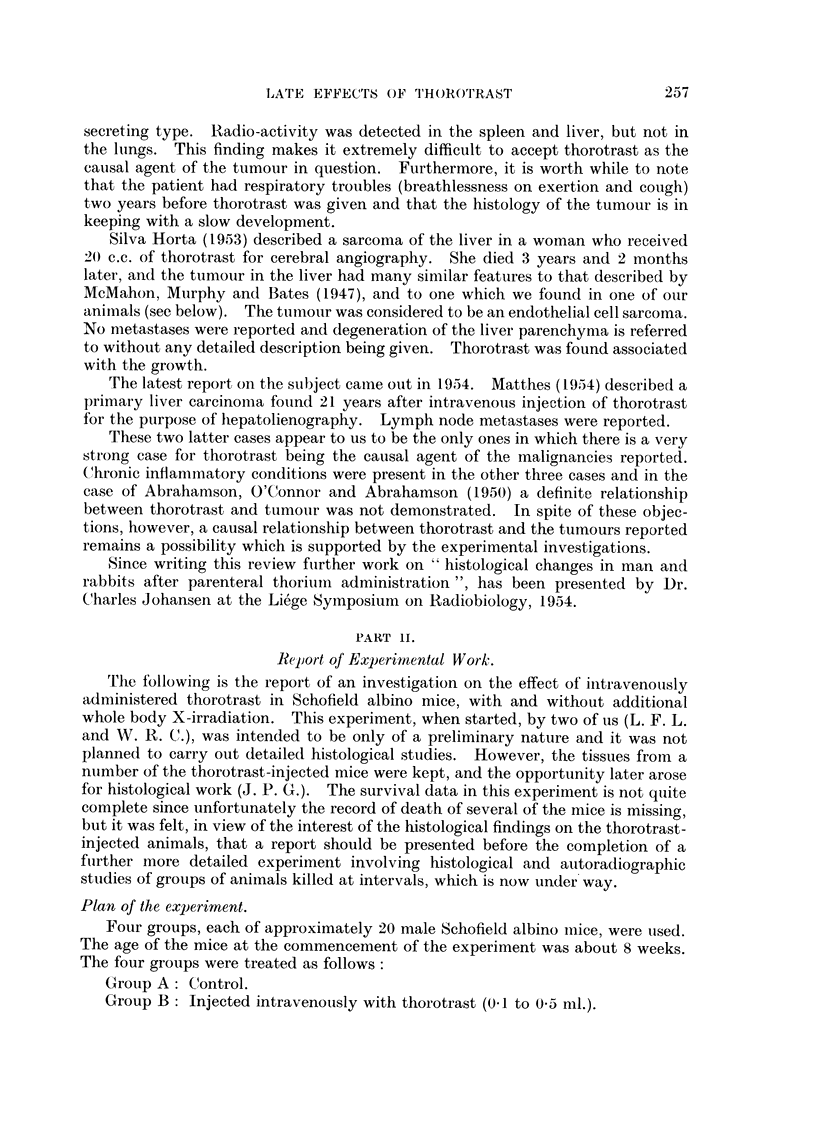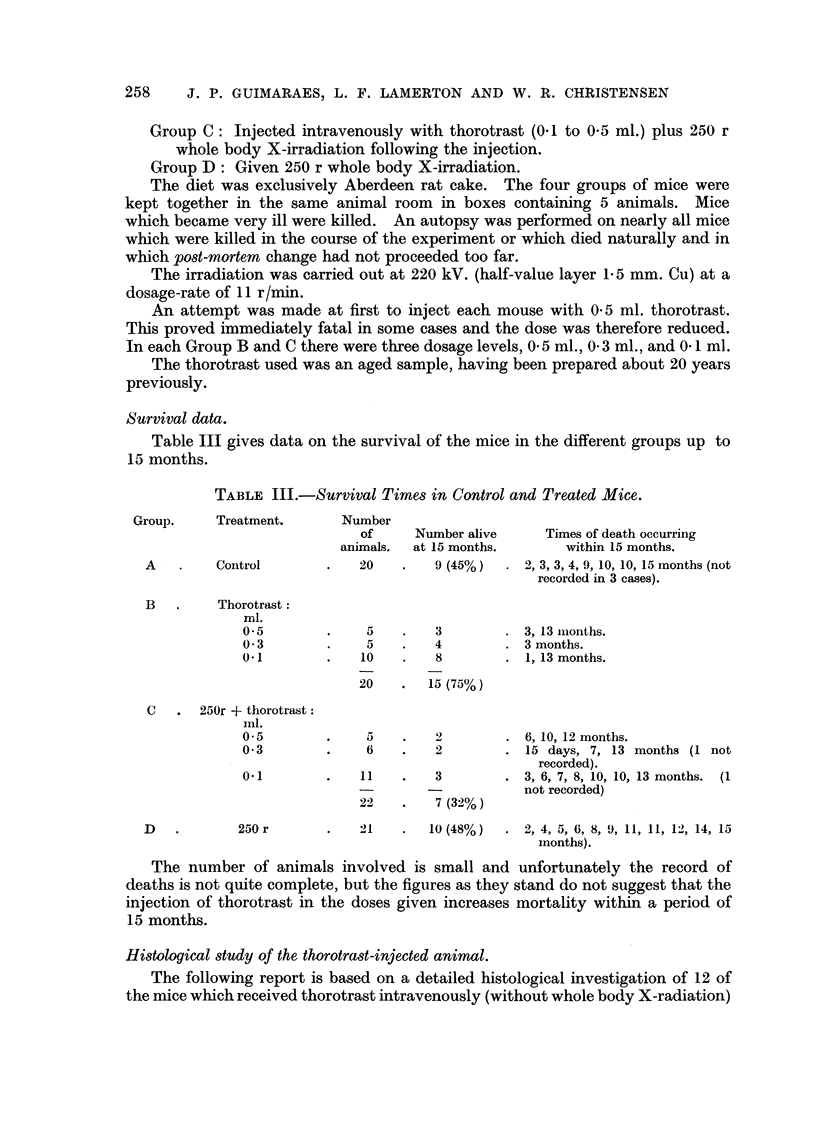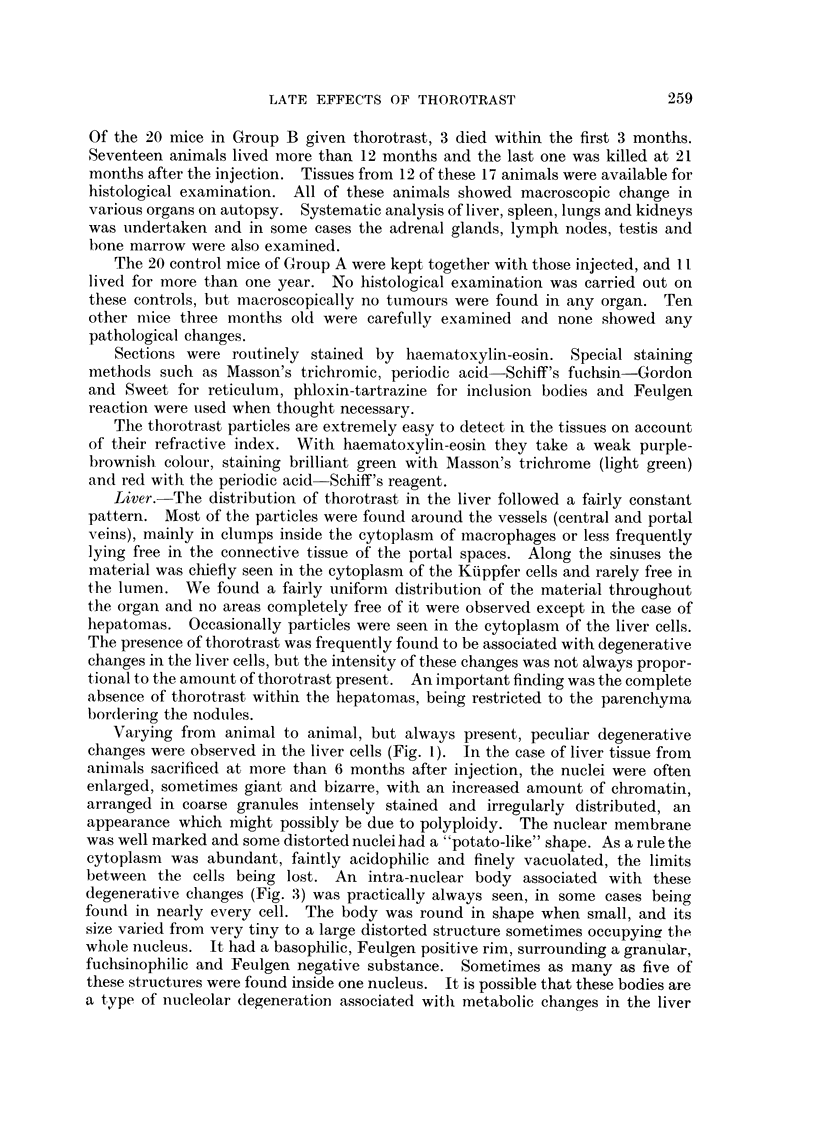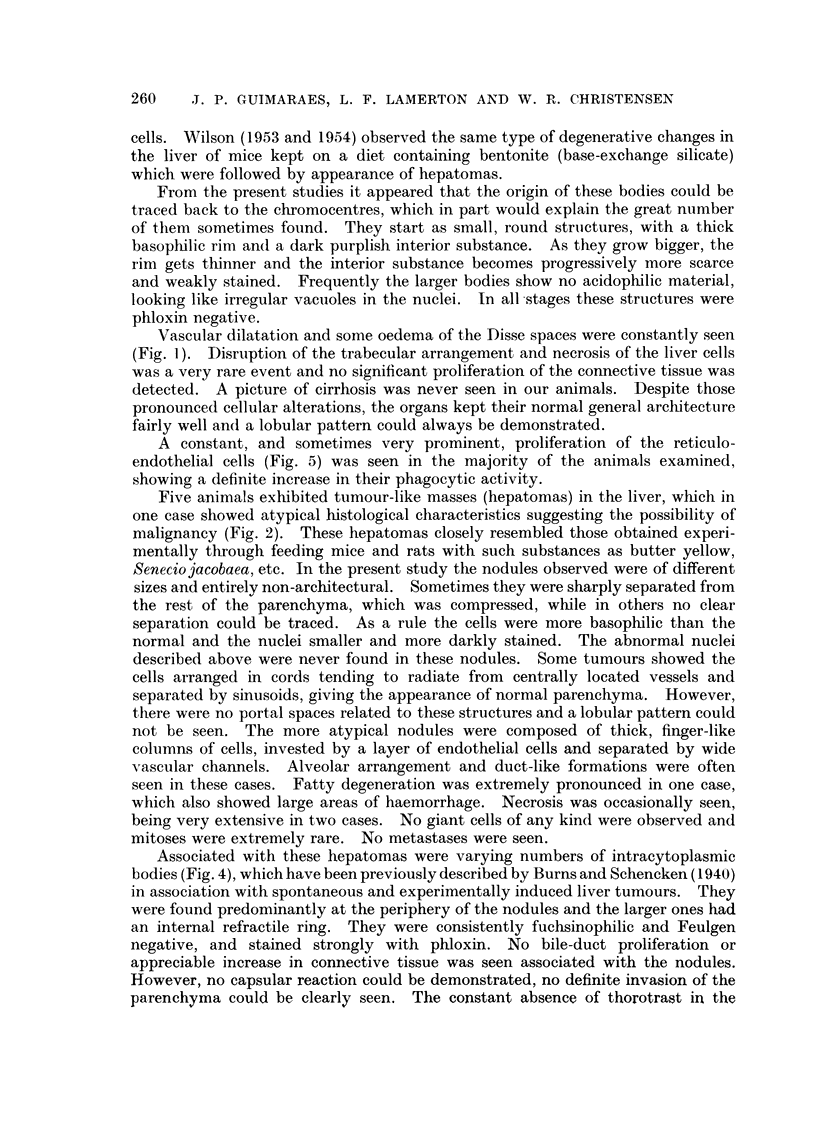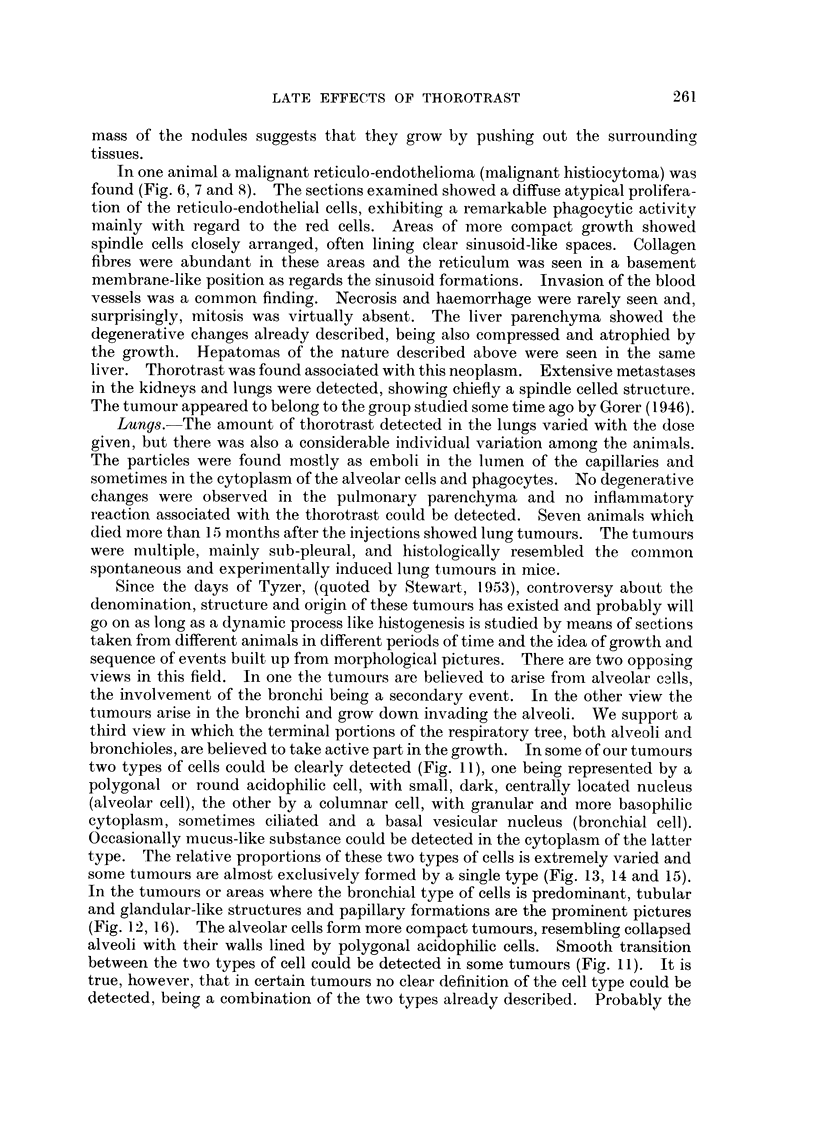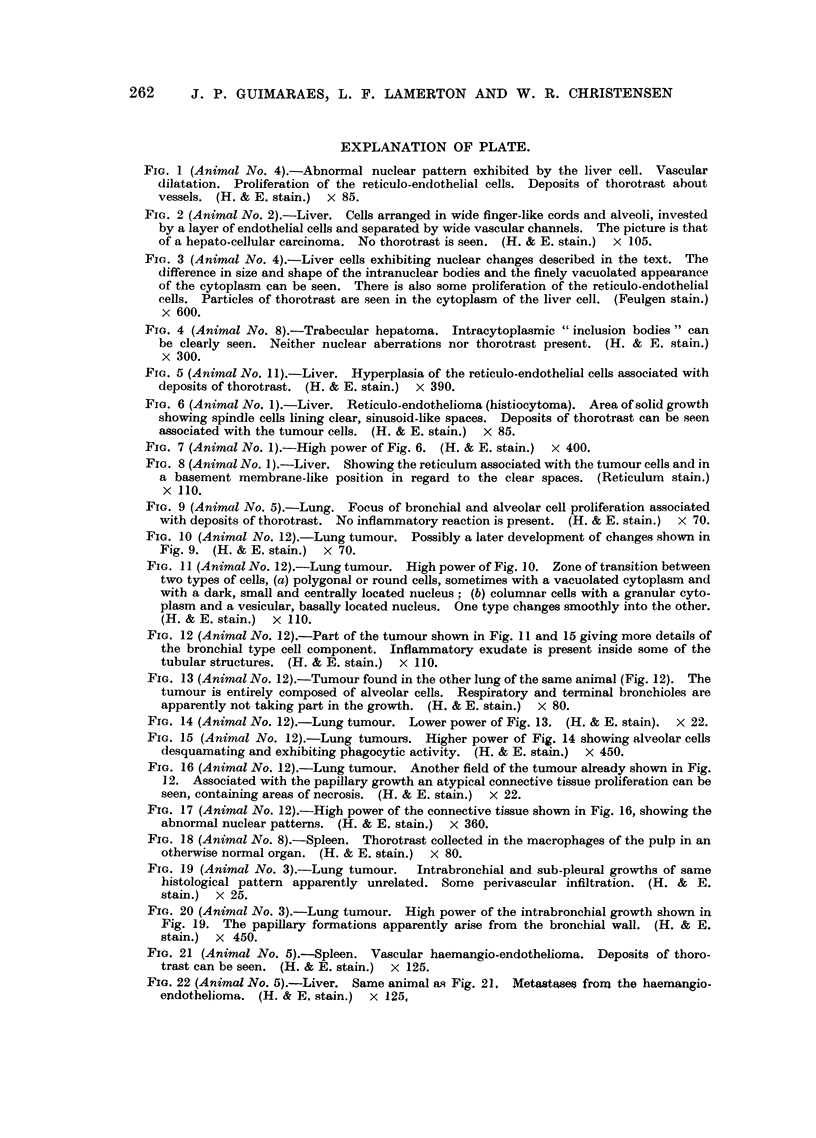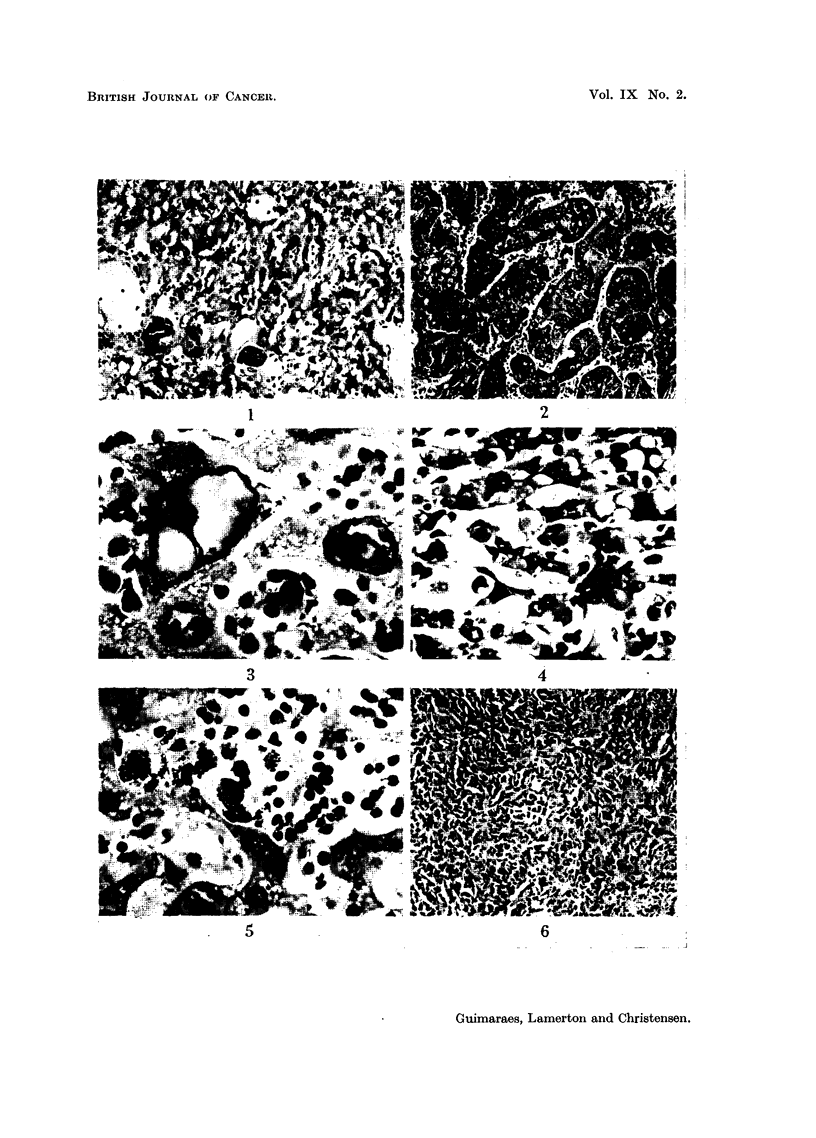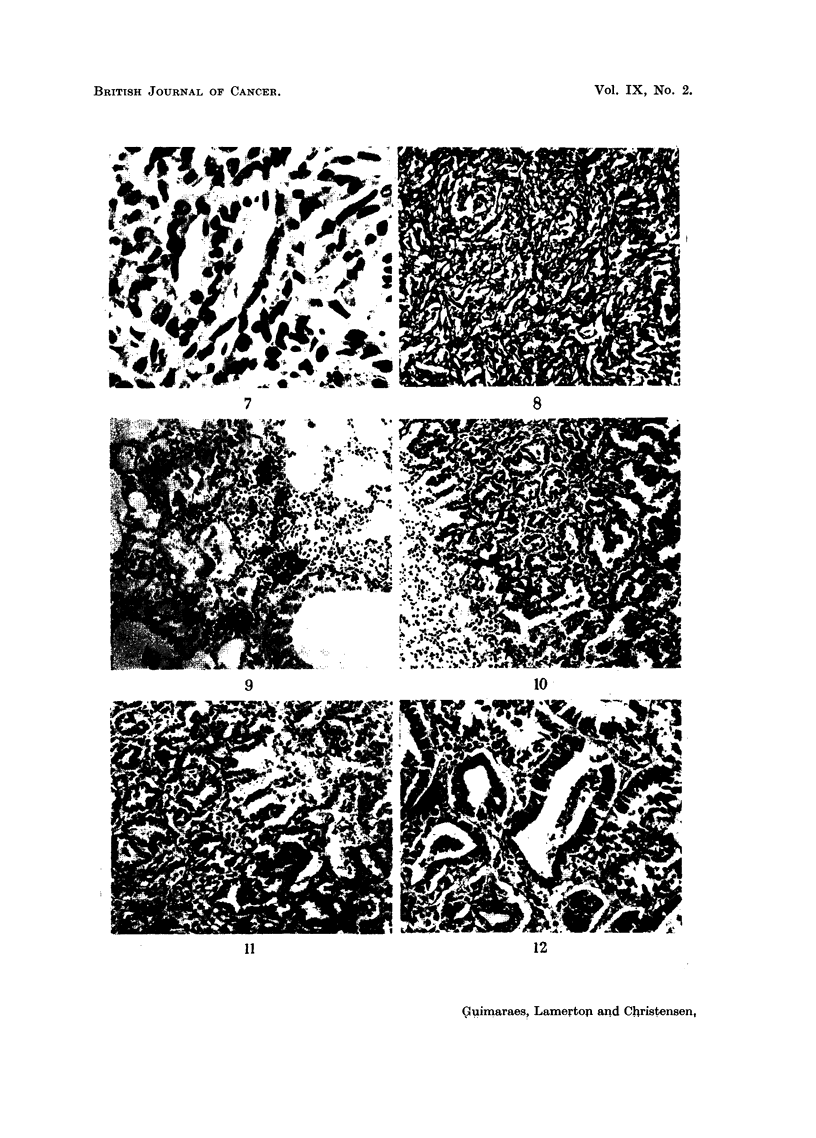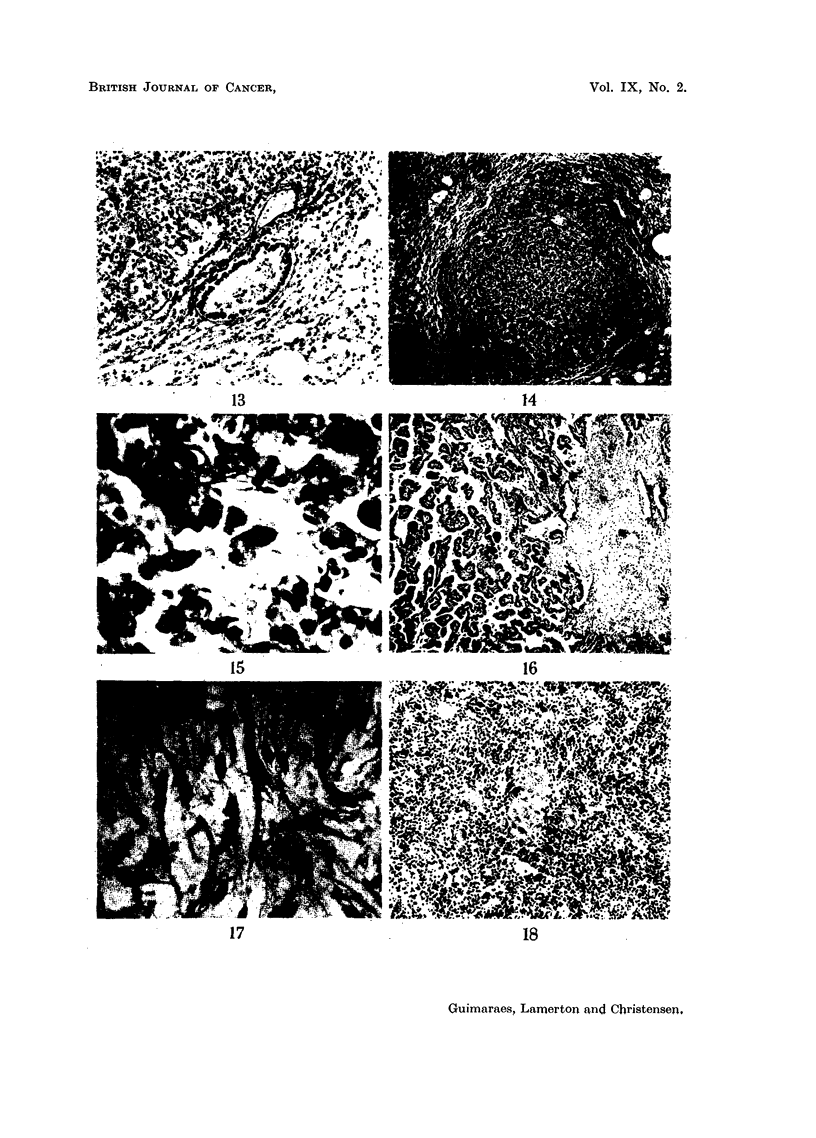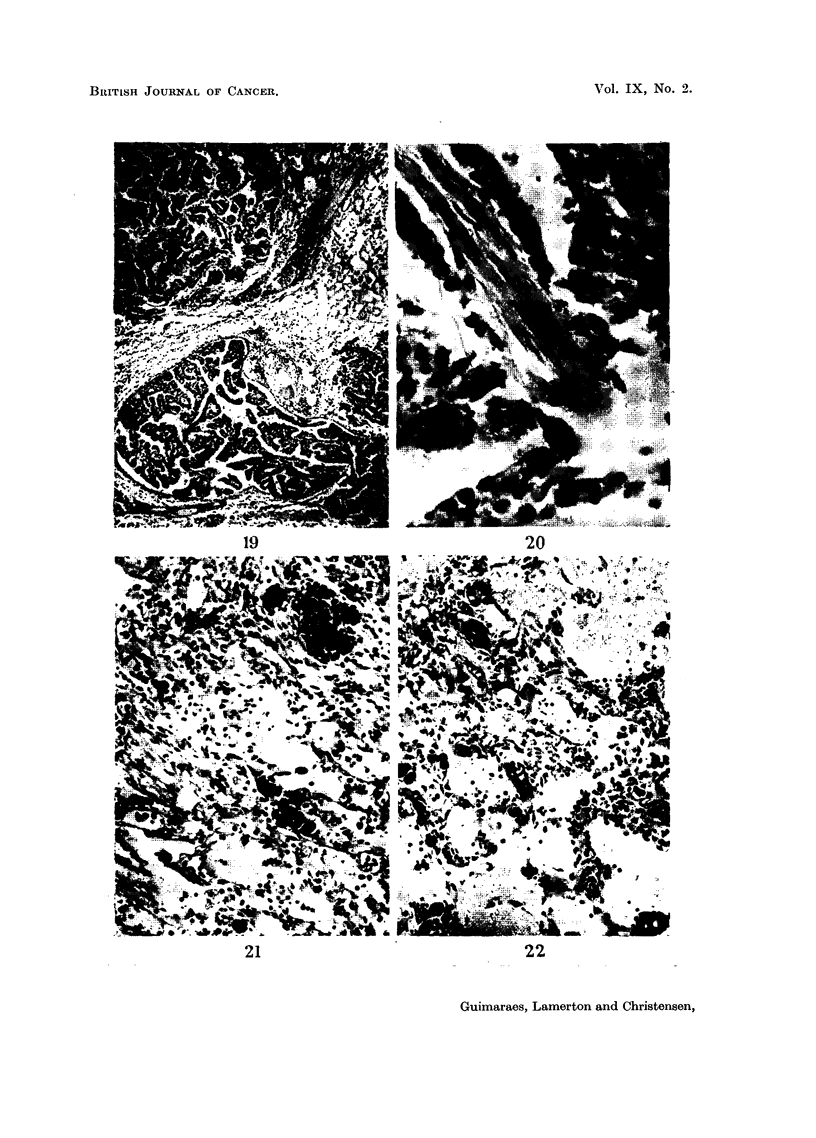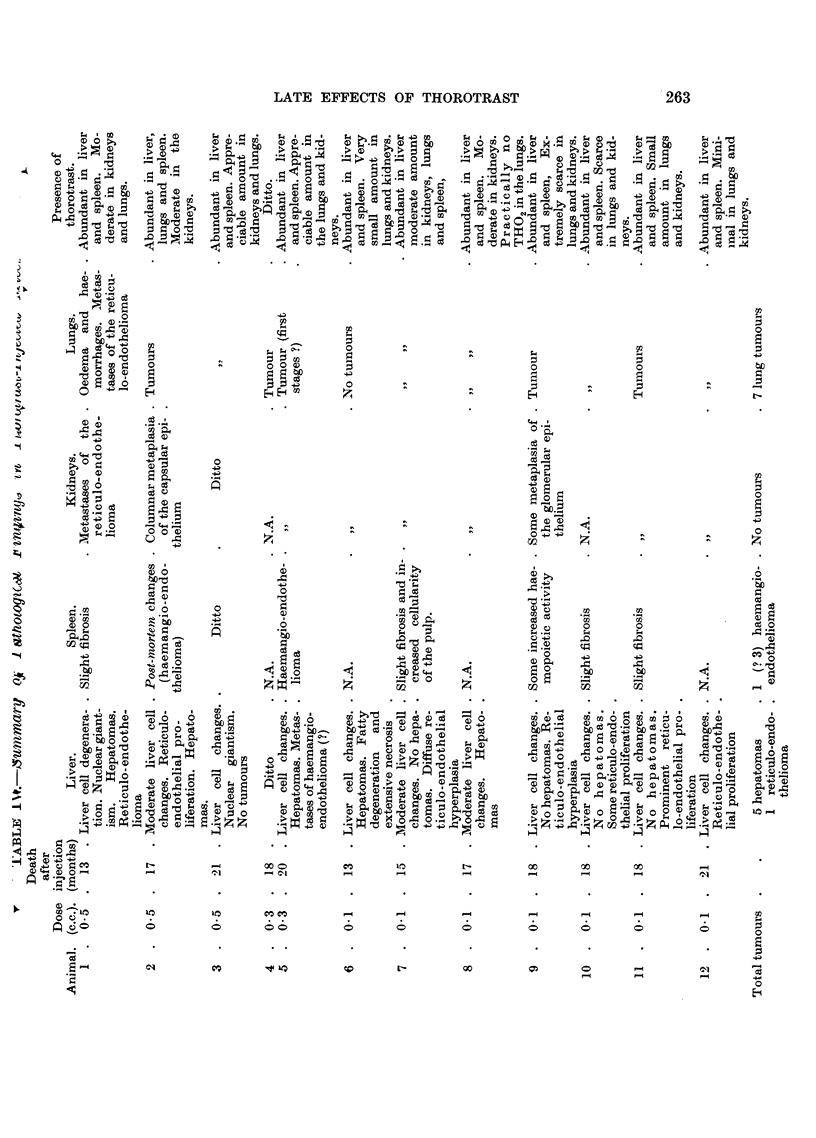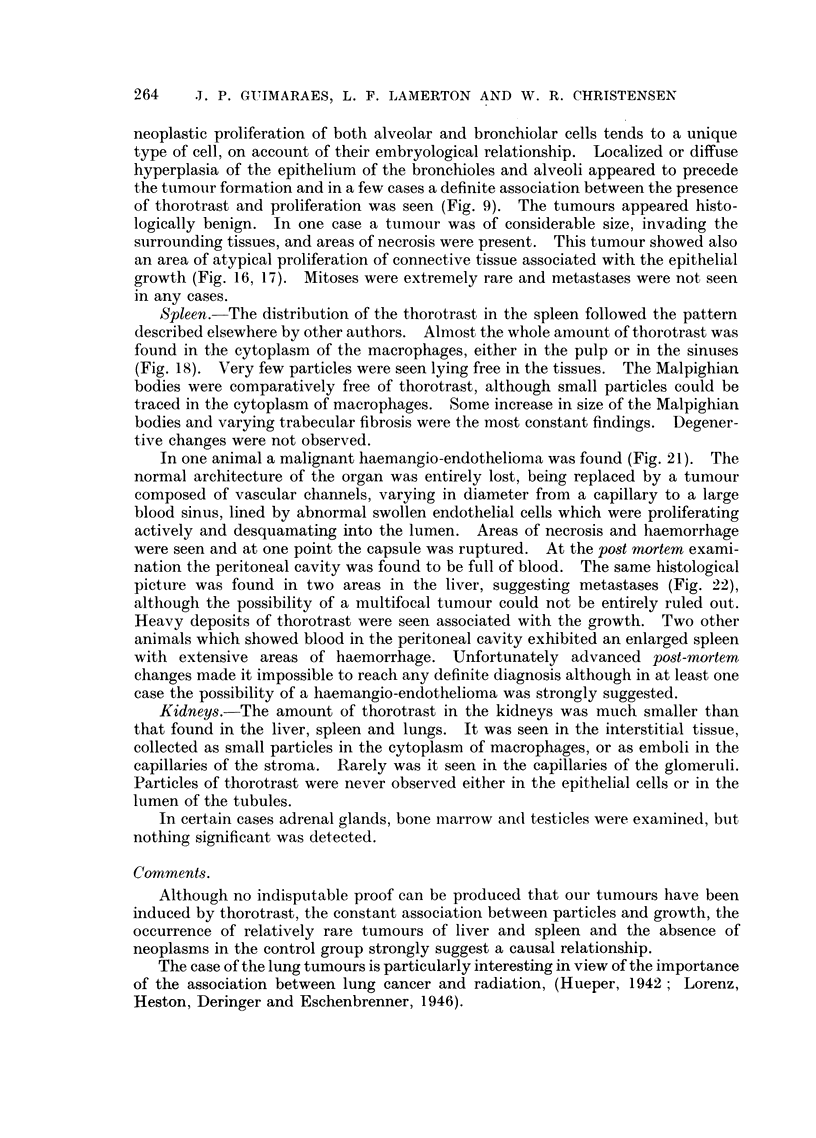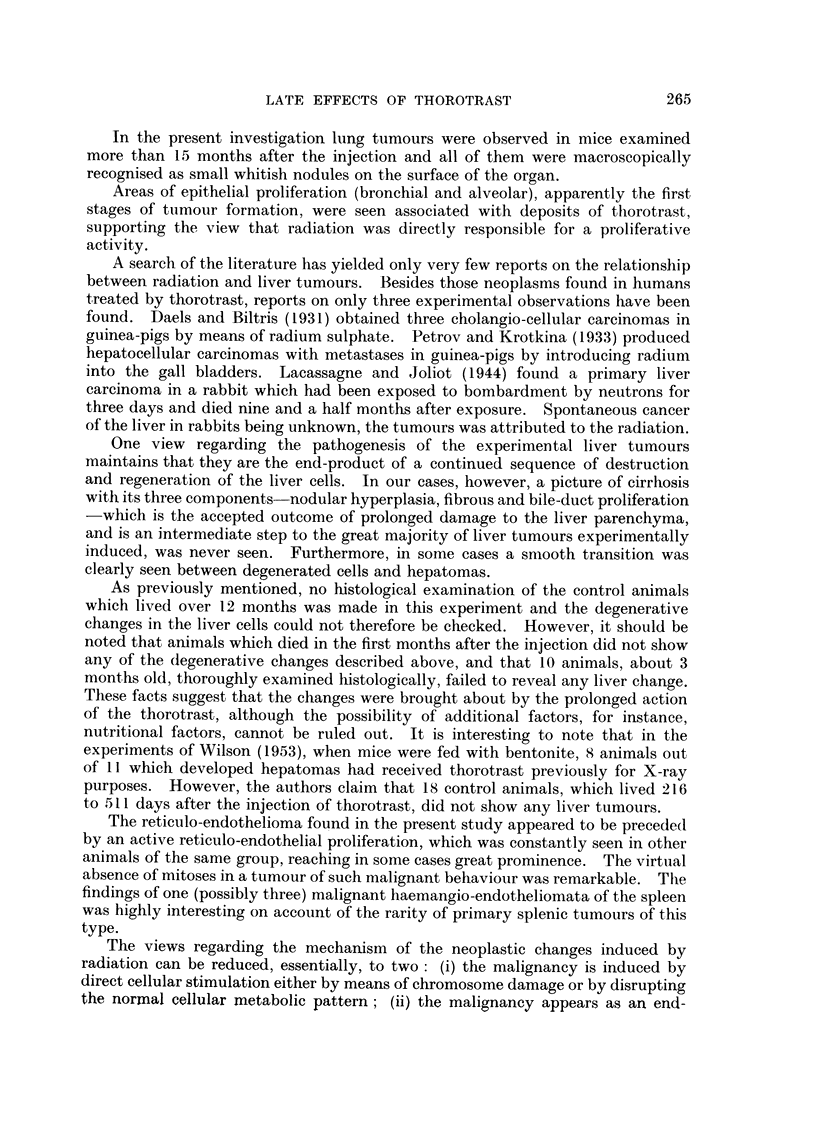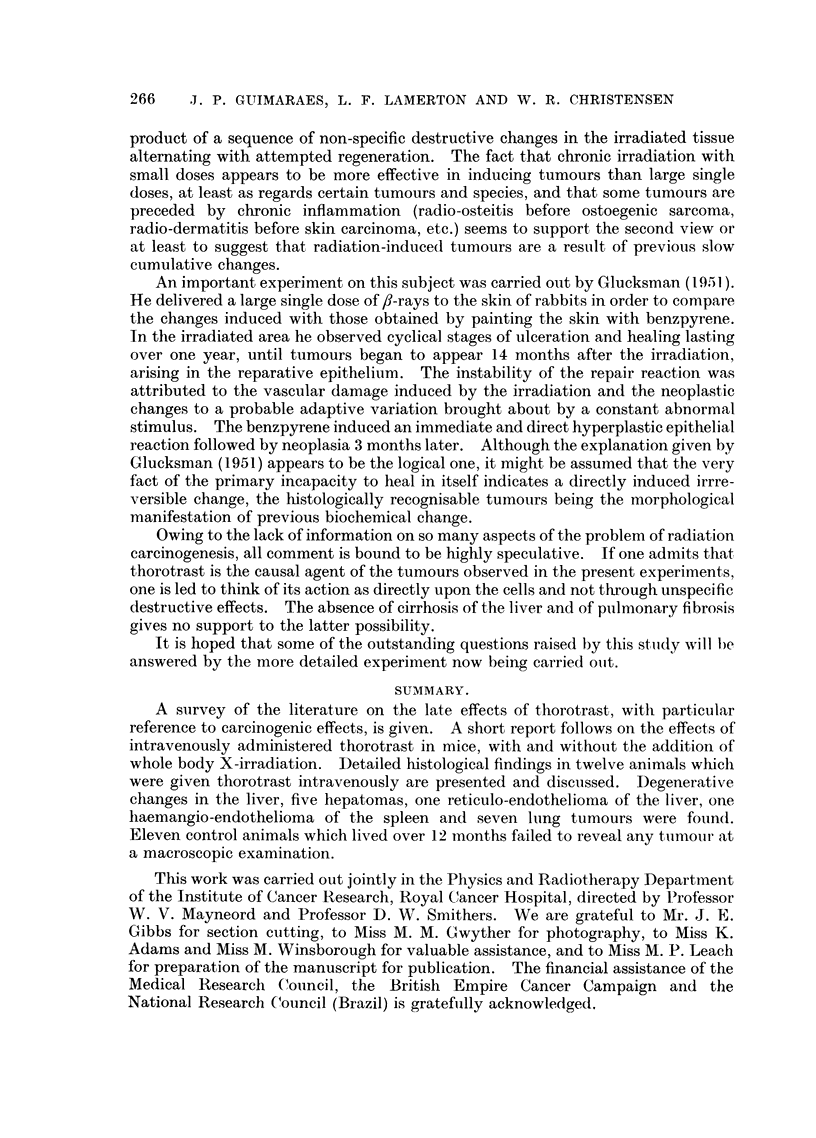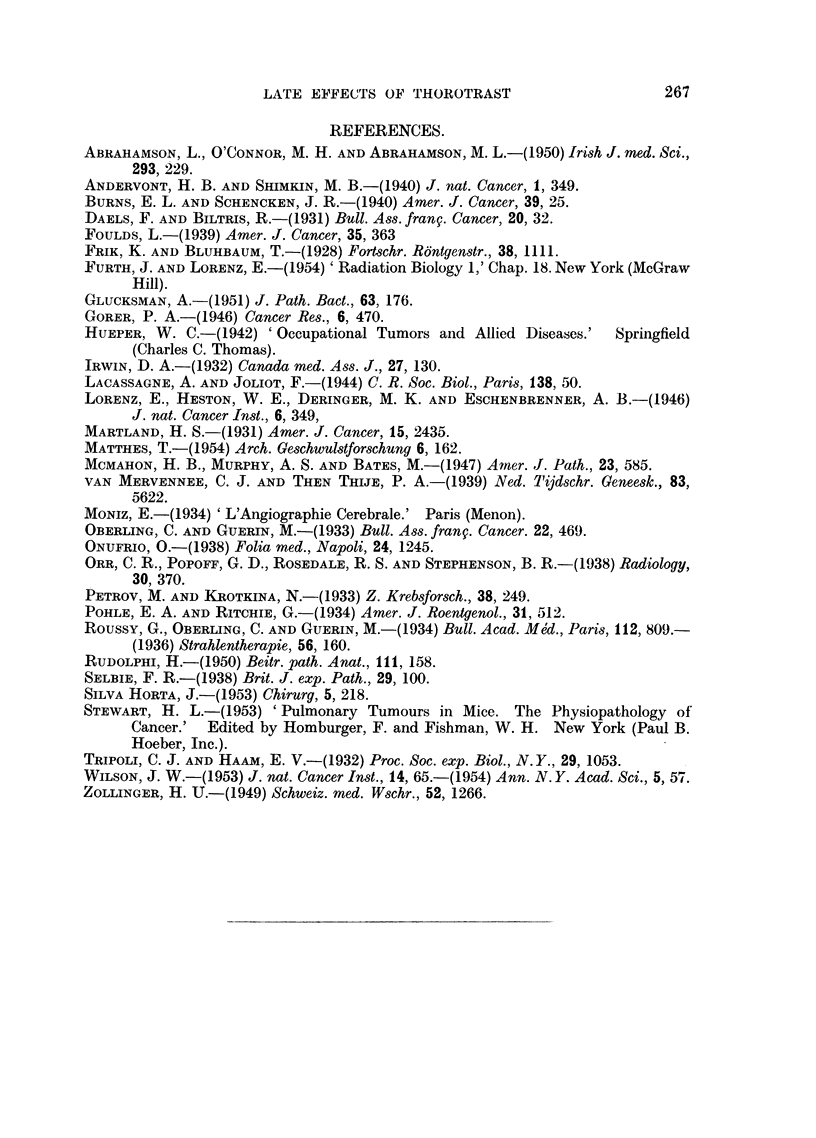# The Late Effects of Thorotrast Administration. A Review and an Experimental study

**DOI:** 10.1038/bjc.1955.22

**Published:** 1955-06

**Authors:** J. P. Guimaraes, L. F. Lamerton, W. R. Christensen

## Abstract

**Images:**


### THE LATE EFFECTS OF THOROTRAST ADMINISTRATION. A REVIEW AND AN EXPERIMENTAL STUDY.

J. P. GUIMARAES, L. F. LAMERTON AND W. R. CHRISTENSEN.

*From the Radiotherapy and Physics Departments, Royal Marsden Hospital, London, S.W.3.*

Received for publication April 26, 1955.

A STUDY of the late effects of intravenously administered thorotrast is one which is of considerable practical importance in view of the past use of this material as a contrast agent in radio-diagnosis, as well as being of great interest in relation to the carcinogenic action of radio-active particulate matter.

Thorotrast is a colloidal solution of thorium dioxide, containing 19–20 per cent of this compound, stabilized by Dextrine (15–20 per cent). The preparation contains methyl-p-hydroxybenzoate (0·15 per cent) as a sterilization agent. In the course of the disintegration of thorium and its daughter elements, $\alpha$, $\beta$ and $\gamma$ radiations are emitted. When injected intravenously, the particles are taken up by the cells of the reticuloendothelial system and, as one would expect, the organs which show the greatest amount of thorotrast after injection are the liver, spleen, bone marrow and lymph nodes. Thorotrast is also found, sometimes in appreciable amounts, in the adrenal glands, kidneys and lungs.

According to McMahon, Murphy and Bates (1947), thorium dioxide was introduced in roentgen diagnosis by Frik and Bluhbaum (1928) who made the first experiments with the substance. Some time later Radt suggested its use in the visualisation of the spleen and liver and developed a suitable solution to be employed in human beings. With the work of Moniz (1934) and his collaborators on the clinical use of thorotrast in cerebral angiography the product became widely adopted.

Thorotrast seemed to be harmless as regards immediate effects, and appeared to be inert as far as biochemical processes were concerned. However, in 1932 the first case of damage (thrombosis) which could be ascribed to thorotrast was reported (Silva Horta, 1953), and in 1947 McMahon, Murphy and Bates published a report of what is thought to be the first case of malignancy related to thorotrast.

The present paper is divided into two parts. The first part is a critical review of the literature on the subject. The second part is the report of an investigation of the effect of intravenously injected thorotrast in the mouse.

### PART I.

### *Review of the Literature.*

From the work of Martland (1931) on the cases of osteogenic sarcoma occurring in Radium dial painters, and the subsequent work of many investigators using artificially produced radio-active materials (Furth and Lorenz, 1954) there is no doubt that a radio-active substance present in the body for a certain length of time may be the source of malignant changes in the tissue.

The introduction of thorotrast in radiological diagnosis aroused suspicions about its possible carcinogenic properties, but most clinicians were sceptical on account of its low activity and the small doses employed. Furthermore, short-term experiments carried out with animals (Tripoli and Haam, 1932 ; Irwin, 1932) seemed to support the belief that thorotrast was harmless under the conditions and in the doses then used.

Oberling and Guerin (1933) injected 1 c.c. of thorotrast intraperitoneally, twice a week for 5 weeks, into rats grafted with Jensen sarcoma, and found that in a number of animals the tumours regressed. Among the rats which had absorbed the tumours, 8 showed peritoneal sarcomatosis and the remaining 2 showed early sarcomatous proliferation. The histology was a little different from that of Jensen sarcoma and attempts to transplant were negative, in spite of the fact that this strain usually yields 100 per cent of takes of Jensen sarcoma.

Pohle and Ritchie (1934) criticised some previous papers in which thorotrast was considered to be harmless, mainly on account of the rather short period of observation of the animals used in the experiments. They carried out an experiment on rabbits in which the animals were observed and studied histologically from 2 to 493 days after the injection. The preparation was injected into the ear vein in doses varying from 0·25 to 0·5 to 1 c.c. per kilogram of body weight. The early changes in the liver were a hydropic degeneration mainly about the central vein and some oedema of the portal spaces. The late findings were scattered areas of atrophy of the parenchyma and slight, but definite, overgrowth of the connective tissue. In the spleen they found fibrosis of the pulp in the majority of the animals. The bone-marrow showed a picture of exhaustion developing two months after the injection. No lesions were found in the lungs, kidneys or adrenals. They did not find any decrease in the amount of thorotrast over the period of observation.

In the same year Roussy, Oberling and Guerin (1934) obtained 8 sarcomas in 15 animals which died between 15 and 17 months after subcutaneous and intraperitoneal injections of thorotrast. The animals received five injections of 1 c.c. given at intervals of 3 and 4 days. Two years later Roussy, Oberling and Guerin (1936) repeated the experiments using doses of 0·5 c.c. and confirmed their previous findings.

Selbie (1938) undertook an experiment using 60 rats and 60 mice, which were injected subcutaneously with two doses of 0·3 c.c. and two doses of 0·1 c.c. respectively. Among the rats, the tumours began to appear at 52 weeks and 25 tumours were found in rats which lived long enough to have them. Twenty-two were spindle cell sarcomas and the other 3 fibromas. Tumours appeared in 58 per cent of the rats. In the mice, the first tumour appeared at 39 weeks, and 9 tumours were obtained : 6 spindle cell sarcomas, an osteosarcoma, a histiocytoma and a capillary angio-endothelioma. Tumours appeared in 43 per cent of the mice. Selbie (1938) suggested that the carcinogenic activity of the thorotrast was probably due not only to its radio-activity but also to the susceptibility of the inflammatory tissue which it produces.

Orr, Popoff, Rosedale and Stephenson (1938) after studying the action of thorotrast injected intravenously in rabbits, concluded that the drug should not be used in human beings because of (1) its lack of elimination from the body, (2) its blockade of the reticulo-endothelial system which may thus adversely affect immunity mechanism, (3) the likelihood that it may profoundly damage the liver

TABLE I.—*Malignancy Related to Thorotrast.* (*Observations in Humans*).

| Author. | Year. | Method. | Dose (c.c.). | Years after. | Type of tumour. | Comments. |
|---|---|---|---|---|---|---|
| McMahon, Murphy and Bates | 1947 | Hepatolienography | 75 | 12 | Endothelial cell sarcoma from liver | Syphilitic back-ground (gumma). |
| Zollinger | 1949 | Retrograde pyelography | 30 | 16 | Spindle cell sarcoma from kidney | Hydronephrosis and chronic pyelonephritis as primary diseases. |
| Rudolphi | 1950 | Dakryocystography | ? | 35 | Squamous carcinoma from eyelid | Chronic inflammation present. |
| Abrahamson, O'Connor and Abrahamson | 1950 | Hepatolienography | 75 | 16 | Alveolar cell tumour from lung | No thorotrast was detected in the lungs. |
| Silva Horta | 1953 | Cerebral angiography | 20 | 3 yrs., 2 mo. | Endothelial cell sarcoma from liver | No comments. |
| Matthes | 1954 | Hepatolienography | 75 | 21 | Liver cell carcinoma | No comments. |

TABLE II.—*Malignancy Related to Thorotrast.* (*Observations in Animals.*)

| Author. | Animal. | Route of injection. | Dose. | Time. | Type of tumour. |
|---|---|---|---|---|---|
| Oberling and Guerin (1933) | Rats | Intraperitoneally | 1 c.c., 10 inj. | After 9 months | Spindle cell sarcomas. |
| Roussy, Oberling and Guerin (1934) | ,, | Intraperitoneally and subcutaneously | 1 c.c. 5 inj. | Between 10th and 17th month | ,, ,, ,, |
| Roussy, Oberling and Guerin (1936) | Rats and mice | Ditto | 0·5 c.c. | Over 1 year | ,, ,, ,, |
| Andervont and Shimkin (1940) | Mice | Subcutaneously | 0·2 and 0·8 c.c. | ,, ,, ,, | Spindle cell sarcomas and haemangiosarcoma. |
| Selbie (1938) | Rats and mice | ,, | 0·3 and 0·1 c.c. | Over 10 months | Spindle cell sarcomas and fibroma. |
| Onufrio (1938) | Rabbits | Intravenously | 6 c.c. | 2 years | Reticulum cell sarcoma. |
| van Mervennee and Then Thije (1939) | ,, | Intravenously (?) | ? | 2 to 3 years | Sarcoma of spleen. |
| Foulds (1939) | Guinea-pig | Subcutaneously (mammary region). | 0·3 c.c. | 3 years | Carcinoma and sarcomata. |

and spleen parenchyma with early and late degenerative changes, and (4) the fact that it is a radioactive substance and undoubtedly has dangerous cumulative effects.

Foulds (1939) obtained 1 carcinoma and 3 sarcomata in 20 guinea-pigs, 3 years after the injection into the mammary region of 0·3 c.c. of thorotrast, repeated each month for 4 months. The carcinoma and 2 of the sarcomas were transplantable.

Andervont and Shimkin (1940) studied the action of thorotrast in Strain A mice which have a high incidence of lung tumours. They concluded that the drug did not increase the incidence of lung tumours in this strain of mouse following intravenous injection of 0·2 to 0·8 c.c.

The first report of a possible connection between thorotrast and malignancy in man was made nearly 20 years after the introduction of the drug for clinical use. In 1947 McMahon, Murphy and Bates published the case of a woman aged 70 who had received 75 c.c. of thorotrast by intravenous injection (three doses of 25 c.c.) 12 years before for diagnostic purposes. She had at this time a gumma of the liver, which was treated, and she made a complete recovery. Twelve years later she developed weakness and pallor and died some time later with the diagnosis of " shock, the result of internal haemorrhage ". Three lesions were demonstrated histologically : ((1) Primary malignant liver tumour with metastases composed of cells resembling Kuppfer cells, which tended to arrange themselves in clusters, as walls of irregular blood sinuses or as walls of blood vessels replacing normal endothelium. Haemorrhage was a very prominent picture in the tumour. (2) Very heavy deposition of thorotrast with severe damage to the liver parenchyma. (3) Scars of healed syphilitic hepatitis with gumma formation. Although the association between tumour and thorotrast appears well demonstrated, the presence of chronic inflammation (syphilis) in the same organ makes it difficult to accept conclusively the aetiological rôle played by the thorotrast in this particular case.

Since this report, five other cases have been published in which thorotrast was thought to be the cause of the tumours found, as follows.:

Zollinger (1949) reported a case of a man of 48 who received 30 c.c. of thorotrast for a retrograde pyelography. Sixteen years later he developed a severe abdominal pain and a spindle cell sarcoma was discovered at operation, in relation to thorotrast deposits. Although Silva Horta (1953) accepts this tumour as definitely due to the action of thorotrast, we rather doubt if the hydronephrosis and chronic pyelonephritis which were the primary disease can be entirely ruled out as conditioning factors.

Rudolphi (1950) published a paper in which he described the case of a man who developed a squamous cell carcinoma of the right lower eyelid 35 years after receiving a preparation of thorium dioxide (0·27 g.) suspended in paraffin, for outlining the lachrymal duct. Chronic inflammation (giant cells) and granulation tissue were found associated with the growth, again bringing doubt about its possible rôle in the genesis of these tumours, although in this case the reaction appears to have been elicited by the presence of the preparation injected.

Abrahamson, O'Connor and Abrahamson (1950) described a lung tumour " with many exceptional, even unique features in a patient who 16 years previously had been injected with 75 c.c. of thorotrast ". Almost every alveoli in both lungs was lined by a tall secretory epithelium yielding mucin into the alveolar space and frequently throwing papillary projections of epithelium into the space. Metastases were found in the hilar lymph nodes and in the pancreas. The authors assumed they were dealing with a primary alveolar tumour of the lung of mucus secreting type. Radio-activity was detected in the spleen and liver, but not in the lungs. This finding makes it extremely difficult to accept thorotrast as the causal agent of the tumour in question. Furthermore, it is worth while to note that the patient had respiratory troubles (breathlessness on exertion and cough) two years before thorotrast was given and that the histology of the tumour is in keeping with a slow development.

Silva Horta (1953) described a sarcoma of the liver in a woman who received 20 c.c. of thorotrast for cerebral angiography. She died 3 years and 2 months later, and the tumour in the liver had many similar features to that described by McMahon, Murphy and Bates (1947), and to one which we found in one of our animals (see below). The tumour was considered to be an endothelial cell sarcoma. No metastases were reported and degeneration of the liver parenchyma is referred to without any detailed description being given. Thorotrast was found associated with the growth.

The latest report on the subject came out in 1954. Matthes (1954) described a primary liver carcinoma found 21 years after intravenous injection of thorotrast for the purpose of hepatolienography. Lymph node metastases were reported.

These two latter cases appear to us to be the only ones in which there is a very strong case for thorotrast being the causal agent of the malignancies reported. Chronic inflammatory conditions were present in the other three cases and in the case of Abrahamson, O'Connor and Abrahamson (1950) a definite relationship between thorotrast and tumour was not demonstrated. In spite of these objections, however, a causal relationship between thorotrast and the tumours reported remains a possibility which is supported by the experimental investigations.

Since writing this review further work on " histological changes in man and rabbits after parenteral thorium administration ", has been presented by Dr. Charles Johansen at the Liége Symposium on Radiobiology, 1954.

### PART II.

### *Report of Experimental Work.*

The following is the report of an investigation on the effect of intravenously administered thorotrast in Schofield albino mice, with and without additional whole body X-irradiation. This experiment, when started, by two of us (L. F. L. and W. R. C.), was intended to be only of a preliminary nature and it was not planned to carry out detailed histological studies. However, the tissues from a number of the thorotrast-injected mice were kept, and the opportunity later arose for histological work (J. P. G.). The survival data in this experiment is not quite complete since unfortunately the record of death of several of the mice is missing, but it was felt, in view of the interest of the histological findings on the thorotrast-injected animals, that a report should be presented before the completion of a further more detailed experiment involving histological and autoradiographic studies of groups of animals killed at intervals, which is now under way.

*Plan of the experiment.*

Four groups, each of approximately 20 male Schofield albino mice, were used. The age of the mice at the commencement of the experiment was about 8 weeks. The four groups were treated as follows :

Group A : Control.

Group B : Injected intravenously with thorotrast (0·1 to 0·5 ml.).

Group C : Injected intravenously with thorotrast (0·1 to 0·5 ml.) plus 250 r whole body X-irradiation following the injection.

Group D : Given 250 r whole body X-irradiation.

The diet was exclusively Aberdeen rat cake. The four groups of mice were kept together in the same animal room in boxes containing 5 animals. Mice which became very ill were killed. An autopsy was performed on nearly all mice which were killed in the course of the experiment or which died naturally and in which *post-mortem* change had not proceeded too far.

The irradiation was carried out at 220 kV. (half-value layer 1·5 mm. Cu) at a dosage-rate of 11 r/min.

An attempt was made at first to inject each mouse with 0·5 ml. thorotrast. This proved immediately fatal in some cases and the dose was therefore reduced. In each Group B and C there were three dosage levels, 0·5 ml., 0·3 ml., and 0·1 ml.

The thorotrast used was an aged sample, having been prepared about 20 years previously.

*Survival data.*

Table III gives data on the survival of the mice in the different groups up to 15 months.

TABLE III.—*Survival Times in Control and Treated Mice.*

| Group. | Treatment. | Number of animals. | Number alive at 15 months. | Times of death occurring within 15 months. |
|---|---|---|---|---|
| A | Control | 20 | 9 (45%) | 2, 3, 3, 4, 9, 10, 10, 15 months (not recorded in 3 cases). |
| B | Thorotrast : ml. | | | |
| | 0·5 | 5 | 3 | 3, 13 months. |
| | 0·3 | 5 | 4 | 3 months. |
| | 0·1 | 10 | 8 | 1, 13 months. |
| | | 20 | 15 (75%) | |
| C | 250r + thorotrast : ml. | | | |
| | 0·5 | 5 | 2 | 6, 10, 12 months. |
| | 0·3 | 6 | 2 | 15 days, 7, 13 months (1 not recorded). |
| | 0·1 | 11 | 3 | 3, 6, 7, 8, 10, 10, 13 months. (1 not recorded) |
| | | 22 | 7 (32%) | |
| D | 250 r | 21 | 10 (48%) | 2, 4, 5, 6, 8, 9, 11, 11, 12, 14, 15 months). |

The number of animals involved is small and unfortunately the record of deaths is not quite complete, but the figures as they stand do not suggest that the injection of thorotrast in the doses given increases mortality within a period of 15 months.

*Histological study of the thorotrast-injected animal.*

The following report is based on a detailed histological investigation of 12 of the mice which received thorotrast intravenously (without whole body X-radiation)

Of the 20 mice in Group B given thorotrast, 3 died within the first 3 months. Seventeen animals lived more than 12 months and the last one was killed at 21 months after the injection. Tissues from 12 of these 17 animals were available for histological examination. All of these animals showed macroscopic change in various organs on autopsy. Systematic analysis of liver, spleen, lungs and kidneys was undertaken and in some cases the adrenal glands, lymph nodes, testis and bone marrow were also examined.

The 20 control mice of Group A were kept together with those injected, and 11 lived for more than one year. No histological examination was carried out on these controls, but macroscopically no tumours were found in any organ. Ten other mice three months old were carefully examined and none showed any pathological changes.

Sections were routinely stained by haematoxylin-eosin. Special staining methods such as Masson's trichromic, periodic acid—Schiff's fuchsin—Gordon and Sweet for reticulum, phloxin-tartrazine for inclusion bodies and Feulgen reaction were used when thought necessary.

The thorotrast particles are extremely easy to detect in the tissues on account of their refractive index. With haematoxylin-eosin they take a weak purple-brownish colour, staining brilliant green with Masson's trichrome (light green) and red with the periodic acid—Schiff's reagent.

*Liver.*—The distribution of thorotrast in the liver followed a fairly constant pattern. Most of the particles were found around the vessels (central and portal veins), mainly in clumps inside the cytoplasm of macrophages or less frequently lying free in the connective tissue of the portal spaces. Along the sinuses the material was chiefly seen in the cytoplasm of the Küppfer cells and rarely free in the lumen. We found a fairly uniform distribution of the material throughout the organ and no areas completely free of it were observed except in the case of hepatomas. Occasionally particles were seen in the cytoplasm of the liver cells. The presence of thorotrast was frequently found to be associated with degenerative changes in the liver cells, but the intensity of these changes was not always proportional to the amount of thorotrast present. An important finding was the complete absence of thorotrast within the hepatomas, being restricted to the parenchyma bordering the nodules.

Varying from animal to animal, but always present, peculiar degenerative changes were observed in the liver cells (Fig. 1). In the case of liver tissue from animals sacrificed at more than 6 months after injection, the nuclei were often enlarged, sometimes giant and bizarre, with an increased amount of chromatin, arranged in coarse granules intensely stained and irregularly distributed, an appearance which might possibly be due to polyploidy. The nuclear membrane was well marked and some distorted nuclei had a "potato-like" shape. As a rule the cytoplasm was abundant, faintly acidophilic and finely vacuolated, the limits between the cells being lost. An intra-nuclear body associated with these degenerative changes (Fig. 3) was practically always seen, in some cases being found in nearly every cell. The body was round in shape when small, and its size varied from very tiny to a large distorted structure sometimes occupying the whole nucleus. It had a basophilic, Feulgen positive rim, surrounding a granular, fuchsinophilic and Feulgen negative substance. Sometimes as many as five of these structures were found inside one nucleus. It is possible that these bodies are a type of nucleolar degeneration associated with metabolic changes in the liver cells. Wilson (1953 and 1954) observed the same type of degenerative changes in the liver of mice kept on a diet containing bentonite (base-exchange silicate) which were followed by appearance of hepatomas.

From the present studies it appeared that the origin of these bodies could be traced back to the chromocentres, which in part would explain the great number of them sometimes found. They start as small, round structures, with a thick basophilic rim and a dark purplish interior substance. As they grow bigger, the rim gets thinner and the interior substance becomes progressively more scarce and weakly stained. Frequently the larger bodies show no acidophilic material, looking like irregular vacuoles in the nuclei. In all stages these structures were phloxin negative.

Vascular dilatation and some oedema of the Disse spaces were constantly seen (Fig. 1). Disruption of the trabecular arrangement and necrosis of the liver cells was a very rare event and no significant proliferation of the connective tissue was detected. A picture of cirrhosis was never seen in our animals. Despite those pronounced cellular alterations, the organs kept their normal general architecture fairly well and a lobular pattern could always be demonstrated.

A constant, and sometimes very prominent, proliferation of the reticulo-endothelial cells (Fig. 5) was seen in the majority of the animals examined, showing a definite increase in their phagocytic activity.

Five animals exhibited tumour-like masses (hepatomas) in the liver, which in one case showed atypical histological characteristics suggesting the possibility of malignancy (Fig. 2). These hepatomas closely resembled those obtained experimentally through feeding mice and rats with such substances as butter yellow, *Senecio jacobaea*, etc. In the present study the nodules observed were of different sizes and entirely non-architectural. Sometimes they were sharply separated from the rest of the parenchyma, which was compressed, while in others no clear separation could be traced. As a rule the cells were more basophilic than the normal and the nuclei smaller and more darkly stained. The abnormal nuclei described above were never found in these nodules. Some tumours showed the cells arranged in cords tending to radiate from centrally located vessels and separated by sinusoids, giving the appearance of normal parenchyma. However, there were no portal spaces related to these structures and a lobular pattern could not be seen. The more atypical nodules were composed of thick, finger-like columns of cells, invested by a layer of endothelial cells and separated by wide vascular channels. Alveolar arrangement and duct-like formations were often seen in these cases. Fatty degeneration was extremely pronounced in one case, which also showed large areas of haemorrhage. Necrosis was occasionally seen, being very extensive in two cases. No giant cells of any kind were observed and mitoses were extremely rare. No metastases were seen.

Associated with these hepatomas were varying numbers of intracytoplasmic bodies (Fig. 4), which have been previously described by Burns and Schencken (1940) in association with spontaneous and experimentally induced liver tumours. They were found predominantly at the periphery of the nodules and the larger ones had an internal refractile ring. They were consistently fuchsinophilic and Feulgen negative, and stained strongly with phloxin. No bile-duct proliferation or appreciable increase in connective tissue was seen associated with the nodules. However, no capsular reaction could be demonstrated, no definite invasion of the parenchyma could be clearly seen. The constant absence of thorotrast in the mass of the nodules suggests that they grow by pushing out the surrounding tissues.

In one animal a malignant reticulo-endothelioma (malignant histiocytoma) was found (Fig. 6, 7 and 8). The sections examined showed a diffuse atypical proliferation of the reticulo-endothelial cells, exhibiting a remarkable phagocytic activity mainly with regard to the red cells. Areas of more compact growth showed spindle cells closely arranged, often lining clear sinusoid-like spaces. Collagen fibres were abundant in these areas and the reticulum was seen in a basement membrane-like position as regards the sinusoid formations. Invasion of the blood vessels was a common finding. Necrosis and haemorrhage were rarely seen and, surprisingly, mitosis was virtually absent. The liver parenchyma showed the degenerative changes already described, being also compressed and atrophied by the growth. Hepatomas of the nature described above were seen in the same liver. Thorotrast was found associated with this neoplasm. Extensive metastases in the kidneys and lungs were detected, showing chiefly a spindle celled structure. The tumour appeared to belong to the group studied some time ago by Gorer (1946).

*Lungs.*—The amount of thorotrast detected in the lungs varied with the dose given, but there was also a considerable individual variation among the animals. The particles were found mostly as emboli in the lumen of the capillaries and sometimes in the cytoplasm of the alveolar cells and phagocytes. No degenerative changes were observed in the pulmonary parenchyma and no inflammatory reaction associated with the thorotrast could be detected. Seven animals which died more than 15 months after the injections showed lung tumours. The tumours were multiple, mainly sub-pleural, and histologically resembled the common spontaneous and experimentally induced lung tumours in mice.

Since the days of Tyzer, (quoted by Stewart, 1953), controversy about the denomination, structure and origin of these tumours has existed and probably will go on as long as a dynamic process like histogenesis is studied by means of sections taken from different animals in different periods of time and the idea of growth and sequence of events built up from morphological pictures. There are two opposing views in this field. In one the tumours are believed to arise from alveolar cells, the involvement of the bronchi being a secondary event. In the other view the tumours arise in the bronchi and grow down invading the alveoli. We support a third view in which the terminal portions of the respiratory tree, both alveoli and bronchioles, are believed to take active part in the growth. In some of our tumours two types of cells could be clearly detected (Fig. 11), one being represented by a polygonal or round acidophilic cell, with small, dark, centrally located nucleus (alveolar cell), the other by a columnar cell, with granular and more basophilic cytoplasm, sometimes ciliated and a basal vesicular nucleus (bronchial cell). Occasionally mucus-like substance could be detected in the cytoplasm of the latter type. The relative proportions of these two types of cells is extremely varied and some tumours are almost exclusively formed by a single type (Fig. 13, 14 and 15). In the tumours or areas where the bronchial type of cells is predominant, tubular and glandular-like structures and papillary formations are the prominent pictures (Fig. 12, 16). The alveolar cells form more compact tumours, resembling collapsed alveoli with their walls lined by polygonal acidophilic cells. Smooth transition between the two types of cell could be detected in some tumours (Fig. 11). It is true, however, that in certain tumours no clear definition of the cell type could be detected, being a combination of the two types already described. Probably the

### EXPLANATION OF PLATE.

FIG. 1 (*Animal No.* 4).—Abnormal nuclear pattern exhibited by the liver cell. Vascular dilatation. Proliferation of the reticulo-endothelial cells. Deposits of thorotrast about vessels. (H. & E. stain.) × 85.

FIG. 2 (*Animal No.* 2).—Liver. Cells arranged in wide finger-like cords and alveoli, invested by a layer of endothelial cells and separated by wide vascular channels. The picture is that of a hepato-cellular carcinoma. No thorotrast is seen. (H. & E. stain.) × 105.

FIG. 3 (*Animal No.* 4).—Liver cells exhibiting nuclear changes described in the text. The difference in size and shape of the intranuclear bodies and the finely vacuolated appearance of the cytoplasm can be seen. There is also some proliferation of the reticulo-endothelial cells. Particles of thorotrast are seen in the cytoplasm of the liver cell. (Feulgen stain.) × 600.

FIG. 4 (*Animal No.* 8).—Trabecular hepatoma. Intracytoplasmic " inclusion bodies " can be clearly seen. Neither nuclear aberrations nor thorotrast present. (H. & E. stain.) × 300.

FIG. 5 (*Animal No.* 11).—Liver. Hyperplasia of the reticulo-endothelial cells associated with deposits of thorotrast. (H. & E. stain.) × 390.

FIG. 6 (*Animal No.* 1).—Liver. Reticulo-endothelioma (histiocytoma). Area of solid growth showing spindle cells lining clear, sinusoid-like spaces. Deposits of thorotrast can be seen associated with the tumour cells. (H. & E. stain.) × 85.

FIG. 7 (*Animal No.* 1).—High power of Fig. 6. (H. & E. stain.) × 400.

FIG. 8 (*Animal No.* 1).—Liver. Showing the reticulum associated with the tumour cells and in a basement membrane-like position in regard to the clear spaces. (Reticulum stain.) × 110.

FIG. 9 (*Animal No.* 5).—Lung. Focus of bronchial and alveolar cell proliferation associated with deposits of thorotrast. No inflammatory reaction is present. (H. & E. stain.) × 70.

FIG. 10 (*Animal No.* 12).—Lung tumour. Possibly a later development of changes shown in Fig. 9. (H. & E. stain.) × 70.

FIG. 11 (*Animal No.* 12).—Lung tumour. High power of Fig. 10. Zone of transition between two types of cells, (*a*) polygonal or round cells, sometimes with a vacuolated cytoplasm and with a dark, small and centrally located nucleus ; (*b*) columnar cells with a granular cytoplasm and a vesicular, basally located nucleus. One type changes smoothly into the other. (H. & E. stain.) × 110.

FIG. 12 (*Animal No.* 12).—Part of the tumour shown in Fig. 11 and 15 giving more details of the bronchial type cell component. Inflammatory exudate is present inside some of the tubular structures. (H. & E. stain.) × 110.

FIG. 13 (*Animal No.* 12).—Tumour found in the other lung of the same animal (Fig. 12). The tumour is entirely composed of alveolar cells. Respiratory and terminal bronchioles are apparently not taking part in the growth. (H. & E. stain.) × 80.

FIG. 14 (*Animal No.* 12).—Lung tumour. Lower power of Fig. 13. (H. & E. stain). × 22.

FIG. 15 (*Animal No.* 12).—Lung tumours. Higher power of Fig. 14 showing alveolar cells desquamating and exhibiting phagocytic activity. (H. & E. stain.) × 450.

FIG. 16 (*Animal No.* 12).—Lung tumour. Another field of the tumour already shown in Fig. 12. Associated with the papillary growth an atypical connective tissue proliferation can be seen, containing areas of necrosis. (H. & E. stain.) × 22.

FIG. 17 (*Animal No.* 12).—High power of the connective tissue shown in Fig. 16, showing the abnormal nuclear patterns. (H. & E. stain.) × 360.

FIG. 18 (*Animal No.* 8).—Spleen. Thorotrast collected in the macrophages of the pulp in an otherwise normal organ. (H. & E. stain.) × 80.

FIG. 19 (*Animal No.* 3).—Lung tumour. Intrabronchial and sub-pleural growths of same histological pattern apparently unrelated. Some perivascular infiltration. (H. & E. stain.) × 25.

FIG. 20 (*Animal No.* 3).—Lung tumour. High power of the intrabronchial growth shown in Fig. 19. The papillary formations apparently arise from the bronchial wall. (H. & E. stain.) × 450.

FIG. 21 (*Animal No.* 5).—Spleen. Vascular haemangio-endothelioma. Deposits of thorotrast can be seen. (H. & E. stain.) × 125.

FIG. 22 (*Animal No.* 5).—Liver. Same animal as Fig. 21. Metastases from the haemangio-endothelioma. (H. & E. stain.) × 125,

1

2

3

4

5

6

7

8

9

10

11

12

Guimaraes, Lamerton and Christensen.

13

14

15

16

17

18

Guimaraes, Lamerton and Christensen.

19

20

21

22

Guimaraes, Lamerton and Christensen,

TABLE IV.—*Summary of Pathological Findings in Thorotrast-Injected Mice.*

| Animal. | Dose (c.c.). | Death after injection (months) | Liver. | Spleen. | Kidneys. | Lungs. | Presence of thorotrast. |
|---|---|---|---|---|---|---|---|
| 1 | 0·5 | 13 | Liver cell degeneration. Nuclear giantism. Hepatomas. Reticulo-endothelioma | Slight fibrosis | Metastases of the reticulo-endothelioma | Oedema and haemorrhages. Metastases of the reticulo-endothelioma | Abundant in liver and spleen. Moderate in kidneys and lungs. |
| 2 | 0·5 | 17 | Moderate liver cell changes. Reticulo-endothelial proliferation. Hepatomas. | *Post-mortem* changes (haemangio-endothelioma) | Columnar metaplasia of the capsular epithelium | Tumours | Abundant in liver, lungs and spleen. Moderate in the kidneys. |
| 3 | 0·5 | 21 | Liver cell changes. Nuclear giantism. No tumours | Ditto | Ditto | ,, | Abundant in liver and spleen. Appreciable amount in kidneys and lungs. |
| 4 | 0·3 | 18 | Ditto | N.A. | N.A. | Tumour | Ditto. |
| 5 | 0·3 | 20 | Liver cell changes. Hepatomas. Metastases of haemangio-endothelioma (?) | Haemangio-endothelioma | ,, | Tumour (first stages ?) | Abundant in liver and spleen. Appreciable amount in the lungs and kidneys. |
| 6 | 0·1 | 13 | Liver cell changes. Hepatomas. Fatty degeneration and extensive necrosis | N.A. | ,, | No tumours | Abundant in liver and spleen. Very small amount in lungs and kidneys. |
| 7 | 0·1 | 15 | Moderate liver cell changes. No hepatomas. Diffuse reticulo-endothelial hyperplasia | Slight fibrosis and increased cellularity of the pulp. | ,, | ,, ,, | Abundant in liver moderate amount in kidneys, lungs and spleen, |
| 8 | 0·1 | 17 | Moderate liver cell changes. Hepatomas | N.A. | ,, | ,, ,, | Abundant in liver and spleen. Moderate in kidneys. Practically no $THO_2$ in the lungs. |
| 9 | 0·1 | 18 | Liver cell changes. No hepatomas. Reticulo-endothelial hyperplasia | Some increased haemopoietic activity | Some metaplasia of the glomerular epithelium | Tumour | Abundant in liver and spleen, Extremely scarce in lungs and kidneys. |
| 10 | 0·1 | 18 | Liver cell changes. No hepatomas. Some reticulo-endothelial proliferation | Slight fibrosis | N.A. | ,, | Abundant in liver and spleen. Scarce in lungs and kidneys. |
| 11 | 0·1 | 18 | Liver cell changes. No hepatomas. Prominent reticulo-endothelial proliferation | Slight fibrosis | ,, | Tumours | Abundant in liver and spleen. Small amount in lungs and kidneys. |
| 12 | 0·1 | 21 | Liver cell changes. Reticulo-endothelial proliferation | N.A. | ,, | ,, | Abundant in liver and spleen. Minimal in lungs and kidneys. |
| Total tumours | | | 5 hepatomas<br>1 reticulo-endothelioma | 1 (? 3) haemangio-endothelioma | No tumours | 7 lung tumours | |

neoplastic proliferation of both alveolar and bronchiolar cells tends to a unique type of cell, on account of their embryological relationship. Localized or diffuse hyperplasia of the epithelium of the bronchioles and alveoli appeared to precede the tumour formation and in a few cases a definite association between the presence of thorotrast and proliferation was seen (Fig. 9). The tumours appeared histologically benign. In one case a tumour was of considerable size, invading the surrounding tissues, and areas of necrosis were present. This tumour showed also an area of atypical proliferation of connective tissue associated with the epithelial growth (Fig. 16, 17). Mitoses were extremely rare and metastases were not seen in any cases.

*Spleen.*—The distribution of the thorotrast in the spleen followed the pattern described elsewhere by other authors. Almost the whole amount of thorotrast was found in the cytoplasm of the macrophages, either in the pulp or in the sinuses (Fig. 18). Very few particles were seen lying free in the tissues. The Malpighian bodies were comparatively free of thorotrast, although small particles could be traced in the cytoplasm of macrophages. Some increase in size of the Malpighian bodies and varying trabecular fibrosis were the most constant findings. Degenertive changes were not observed.

In one animal a malignant haemangio-endothelioma was found (Fig. 21). The normal architecture of the organ was entirely lost, being replaced by a tumour composed of vascular channels, varying in diameter from a capillary to a large blood sinus, lined by abnormal swollen endothelial cells which were proliferating actively and desquamating into the lumen. Areas of necrosis and haemorrhage were seen and at one point the capsule was ruptured. At the *post mortem* examination the peritoneal cavity was found to be full of blood. The same histological picture was found in two areas in the liver, suggesting metastases (Fig. 22), although the possibility of a multifocal tumour could not be entirely ruled out. Heavy deposits of thorotrast were seen associated with the growth. Two other animals which showed blood in the peritoneal cavity exhibited an enlarged spleen with extensive areas of haemorrhage. Unfortunately advanced *post-mortem* changes made it impossible to reach any definite diagnosis although in at least one case the possibility of a haemangio-endothelioma was strongly suggested.

*Kidneys.*—The amount of thorotrast in the kidneys was much smaller than that found in the liver, spleen and lungs. It was seen in the interstitial tissue, collected as small particles in the cytoplasm of macrophages, or as emboli in the capillaries of the stroma. Rarely was it seen in the capillaries of the glomeruli. Particles of thorotrast were never observed either in the epithelial cells or in the lumen of the tubules.

In certain cases adrenal glands, bone marrow and testicles were examined, but nothing significant was detected.

*Comments.*

Although no indisputable proof can be produced that our tumours have been induced by thorotrast, the constant association between particles and growth, the occurrence of relatively rare tumours of liver and spleen and the absence of neoplasms in the control group strongly suggest a causal relationship.

The case of the lung tumours is particularly interesting in view of the importance of the association between lung cancer and radiation, (Hueper, 1942; Lorenz, Heston, Deringer and Eschenbrenner, 1946).

In the present investigation lung tumours were observed in mice examined more than 15 months after the injection and all of them were macroscopically recognised as small whitish nodules on the surface of the organ.

Areas of epithelial proliferation (bronchial and alveolar), apparently the first stages of tumour formation, were seen associated with deposits of thorotrast, supporting the view that radiation was directly responsible for a proliferative activity.

A search of the literature has yielded only very few reports on the relationship between radiation and liver tumours. Besides those neoplasms found in humans treated by thorotrast, reports on only three experimental observations have been found. Daels and Biltris (1931) obtained three cholangio-cellular carcinomas in guinea-pigs by means of radium sulphate. Petrov and Krotkina (1933) produced hepatocellular carcinomas with metastases in guinea-pigs by introducing radium into the gall bladders. Lacassagne and Joliot (1944) found a primary liver carcinoma in a rabbit which had been exposed to bombardment by neutrons for three days and died nine and a half months after exposure. Spontaneous cancer of the liver in rabbits being unknown, the tumours was attributed to the radiation.

One view regarding the pathogenesis of the experimental liver tumours maintains that they are the end-product of a continued sequence of destruction and regeneration of the liver cells. In our cases, however, a picture of cirrhosis with its three components—nodular hyperplasia, fibrous and bile-duct proliferation —which is the accepted outcome of prolonged damage to the liver parenchyma, and is an intermediate step to the great majority of liver tumours experimentally induced, was never seen. Furthermore, in some cases a smooth transition was clearly seen between degenerated cells and hepatomas.

As previously mentioned, no histological examination of the control animals which lived over 12 months was made in this experiment and the degenerative changes in the liver cells could not therefore be checked. However, it should be noted that animals which died in the first months after the injection did not show any of the degenerative changes described above, and that 10 animals, about 3 months old, thoroughly examined histologically, failed to reveal any liver change. These facts suggest that the changes were brought about by the prolonged action of the thorotrast, although the possibility of additional factors, for instance, nutritional factors, cannot be ruled out. It is interesting to note that in the experiments of Wilson (1953), when mice were fed with bentonite, 8 animals out of 11 which developed hepatomas had received thorotrast previously for X-ray purposes. However, the authors claim that 18 control animals, which lived 216 to 511 days after the injection of thorotrast, did not show any liver tumours.

The reticulo-endothelioma found in the present study appeared to be preceded by an active reticulo-endothelial proliferation, which was constantly seen in other animals of the same group, reaching in some cases great prominence. The virtual absence of mitoses in a tumour of such malignant behaviour was remarkable. The findings of one (possibly three) malignant haemangio-endotheliomata of the spleen was highly interesting on account of the rarity of primary splenic tumours of this type.

The views regarding the mechanism of the neoplastic changes induced by radiation can be reduced, essentially, to two : (i) the malignancy is induced by direct cellular stimulation either by means of chromosome damage or by disrupting the normal cellular metabolic pattern ; (ii) the malignancy appears as an endproduct of a sequence of non-specific destructive changes in the irradiated tissue alternating with attempted regeneration. The fact that chronic irradiation with small doses appears to be more effective in inducing tumours than large single doses, at least as regards certain tumours and species, and that some tumours are preceded by chronic inflammation (radio-osteitis before ostoegenic sarcoma, radio-dermatitis before skin carcinoma, etc.) seems to support the second view or at least to suggest that radiation-induced tumours are a result of previous slow cumulative changes.

An important experiment on this subject was carried out by Glucksman (1951). He delivered a large single dose of $\beta$-rays to the skin of rabbits in order to compare the changes induced with those obtained by painting the skin with benzpyrene. In the irradiated area he observed cyclical stages of ulceration and healing lasting over one year, until tumours began to appear 14 months after the irradiation, arising in the reparative epithelium. The instability of the repair reaction was attributed to the vascular damage induced by the irradiation and the neoplastic changes to a probable adaptive variation brought about by a constant abnormal stimulus. The benzpyrene induced an immediate and direct hyperplastic epithelial reaction followed by neoplasia 3 months later. Although the explanation given by Glucksman (1951) appears to be the logical one, it might be assumed that the very fact of the primary incapacity to heal in itself indicates a directly induced irreversible change, the histologically recognisable tumours being the morphological manifestation of previous biochemical change.

Owing to the lack of information on so many aspects of the problem of radiation carcinogenesis, all comment is bound to be highly speculative. If one admits that thorotrast is the causal agent of the tumours observed in the present experiments, one is led to think of its action as directly upon the cells and not through unspecific destructive effects. The absence of cirrhosis of the liver and of pulmonary fibrosis gives no support to the latter possibility.

It is hoped that some of the outstanding questions raised by this study will be answered by the more detailed experiment now being carried out.

SUMMARY.

A survey of the literature on the late effects of thorotrast, with particular reference to carcinogenic effects, is given. A short report follows on the effects of intravenously administered thorotrast in mice, with and without the addition of whole body X-irradiation. Detailed histological findings in twelve animals which were given thorotrast intravenously are presented and discussed. Degenerative changes in the liver, five hepatomas, one reticulo-endothelioma of the liver, one haemangio-endothelioma of the spleen and seven lung tumours were found. Eleven control animals which lived over 12 months failed to reveal any tumour at a macroscopic examination.

This work was carried out jointly in the Physics and Radiotherapy Department of the Institute of Cancer Research, Royal Cancer Hospital, directed by Professor W. V. Mayneord and Professor D. W. Smithers. We are grateful to Mr. J. E. Gibbs for section cutting, to Miss M. M. Gwyther for photography, to Miss K. Adams and Miss M. Winsborough for valuable assistance, and to Miss M. P. Leach for preparation of the manuscript for publication. The financial assistance of the Medical Research Council, the British Empire Cancer Campaign and the National Research Council (Brazil) is gratefully acknowledged.

### REFERENCES.

Abrahamson, L., O'Connor, M. H. and Abrahamson, M. L.—(1950) *Irish J. med. Sci.*, **293**, 229.
Andervont, H. B. and Shimkin, M. B.—(1940) *J. nat. Cancer*, **1**, 349.
Burns, E. L. and Schencken, J. R.—(1940) *Amer. J. Cancer*, **39**, 25.
Daels, F. and Biltris, R.—(1931) *Bull. Ass. franç. Cancer*, **20**, 32.
Foulds, L.—(1939) *Amer. J. Cancer*, **35**, 363
Frik, K. and Bluhbaum, T.—(1928) *Fortschr. Röntgenstr.*, **38**, 1111.
Furth, J. and Lorenz, E.—(1954) ' Radiation Biology 1,' Chap. 18. New York (McGraw Hill).
Glucksman, A.—(1951) *J. Path. Bact.*, **63**, 176.
Gorer, P. A.—(1946) *Cancer Res.*, **6**, 470.
Hueper, W. C.—(1942) ' Occupational Tumors and Allied Diseases.' Springfield (Charles C. Thomas).
Irwin, D. A.—(1932) *Canada med. Ass. J.*, **27**, 130.
Lacassagne, A. and Joliot, F.—(1944) *C. R. Soc. Biol., Paris*, **138**, 50.
Lorenz, E., Heston, W. E., Deringer, M. K. and Eschenbrenner, A. B.—(1946) *J. nat. Cancer Inst.*, **6**, 349,
Martland, H. S.—(1931) *Amer. J. Cancer*, **15**, 2435.
Matthes, T.—(1954) *Arch. Geschwulstforschung* **6**, 162.
McMahon, H. B., Murphy, A. S. and Bates, M.—(1947) *Amer. J. Path.*, **23**, 585.
van Mervennee, C. J. and Then Thije, P. A.—(1939) *Ned. Tijdschr. Geneesk.*, **83**, 5622.
Moniz, E.—(1934) ' L'Angiographie Cerebrale.' Paris (Menon).
Oberling, C. and Guerin, M.—(1933) *Bull. Ass. franç. Cancer*. **22**, 469.
Onufrio, O.—(1938) *Folia med., Napoli*, **24**, 1245.
Orr, C. R., Popoff, G. D., Rosedale, R. S. and Stephenson, B. R.—(1938) *Radiology*, **30**, 370.
Petrov, M. and Krotkina, N.—(1933) *Z. Krebsforsch.*, **38**, 249.
Pohle, E. A. and Ritchie, G.—(1934) *Amer. J. Roentgenol.*, **31**, 512.
Roussy, G., Oberling, C. and Guerin, M.—(1934) *Bull. Acad. Méd., Paris*, **112**, 809.—(1936) *Strahlentherapie*, **56**, 160.
Rudolphi, H.—(1950) *Beitr. path. Anat.*, **111**, 158.
Selbie, F. R.—(1938) *Brit. J. exp. Path.*, **29**, 100.
Silva Horta, J.—(1953) *Chirurg*, **5**, 218.
Stewart, H. L.—(1953) ' Pulmonary Tumours in Mice. The Physiopathology of Cancer.' Edited by Homburger, F. and Fishman, W. H. New York (Paul B. Hoeber, Inc.).
Tripoli, C. J. and Haam, E. V.—(1932) *Proc. Soc. exp. Biol., N.Y.*, **29**, 1053.
Wilson, J. W.—(1953) *J. nat. Cancer Inst.*, **14**, 65.—(1954) *Ann. N.Y. Acad. Sci.*, **5**, 57.
Zollinger, H. U.—(1949) *Schweiz. med. Wschr.*, **52**, 1266.